# Nanophotonic route to control electron behaviors in 2D materials

**DOI:** 10.1515/nanoph-2024-0074

**Published:** 2024-05-10

**Authors:** DongJun Kang, Chibuzo Onwukaeme, KiJeong Park, KyeongPyo Jeon, Han-Youl Ryu, SeokJae Yoo

**Affiliations:** 26718 Inha University, Incheon, Republic of Korea

**Keywords:** Purcell effect, strong coupling, optical cavity, 2D materials, Dirac materials

## Abstract

Two-dimensional (2D) Dirac materials, e.g., graphene and transition metal dichalcogenides (TMDs), are one-atom-thick monolayers whose electronic behaviors are described by the Dirac equation. These materials serve not only as test beds for novel quantum physics but also as promising constituents for nanophotonic devices. This review provides a brief overview of the recent effort to control Dirac electron behaviors using nanophotonics. We introduce a principle of light-2D Dirac matter interaction to offer a design guide for 2D Dirac material–based nanophotonic devices. We also discuss opportunities for coupling nanophotonics with externally perturbed 2D materials.

## 1 Introduction

Two-dimensional (2D) materials, one-atom-thick crystals, have unique properties that cannot be found in their bulk counterparts. Quantum behaviors of electrons in some 2D materials, e.g., graphene, transition metal dichalcogenides (TMDs), and hexagonal boron nitrides (hBNs), follow the Dirac equation rather than the Schrodinger equation [1], [2]. In the past two decades after the pioneering works on the exfoliation of graphene and the measurement of its Dirac fermionic behaviors [3], [4], [5], the 2D Dirac materials have been of huge interest in scientific communities because they can play the role of testbeds for novel physics, such as the half-integer quantum Hall effect [6], the ballistic transport [7], and graphene plasmonics [8], [9], [10].

2D gapped Dirac materials with a honeycomb lattice can have multiple bandgaps at different positions in the momentum space, the so-called valleys. Especially, 2D TMDs have direct bandgaps in visible frequencies, allowing optical accesses to the valleys, while strong spin–orbit coupling (SOC) provides the spin-valley locking [11]. The valleys in 2D TMDs can be selectively populated, i.e., the valley polarization, by circularly polarized optical pumping [11], [12], [13], [14] and the spin injection from the ferromagnetic electrodes [15]. The valley degree of freedom in the 2D Dirac materials gave birth to a new field to process information using valleys and spins, namely valleytronics [16].

Valley-contrasting physics, together with the direct bandgaps in 2D TMDs, have intrigued nanophotonic efforts. In a practical point of view, the 2D nature of the materials is compatible with lithographically defined nanosurfaces such as plasmonic nanostructures and metasurfaces. 2D materials are also easy to exfoliate and transfer at the lab scale. Triggered by the advantages of 2D materials, it has been demonstrated that the excitonic transitions photoluminescence (PL) in 2D TMDs can be readily controlled by the Purcell effect [17], [18], [19], [20], the chiral Purcell effect [21], [22], the strong coupling [23], [24], [25], and the plasmonic routing [26]. These approaches envision possibilities and opportunities in optoelectronic and optical information processing devices based on nanophotonically controlled 2D Dirac materials.

Due to growing interest in nanophotonic structure/2D material systems, many review articles have been published, offering comprehensive lists of important works on nanophotonics using 2D materials [27], [28], [29], [30], [31]. While some focus on specific topics, such as interaction between circularly polarized light and 2D materials [31], coupling to the Mie resonance of nanoparticles [27], and strong coupling [29], we aim to discuss the nanophotonic control of electron behaviors in 2D Dirac materials, its underlying principles, and future directions.

In this review, we introduce efforts to control electron behaviors in nanophotonics-coupled 2D materials and give our perspective for future research. In Section 2, we first provide a comprehensive overview of recent nanophotonic research using 2D materials. Then, in Section 3, we discuss a hierarchical structure of light, i.e., momentum-energy and spin angular momentum-helicity, and its chiral coupling to 2D Dirac materials. We also discuss the analogy and distinction between 2D materials and chiral molecules when they are coupled to circularly polarized light in a chiral manner. In Section 4, we provide our perspectives by listing the unique properties of 2D materials that can be controlled by nanophotonics. We expect that our review can promote nanophotonic approaches to control electron behaviors in 2D materials and introduce novel optoelectronic applications.

## 2 Overview: nanophotonic control of electron behaviors in 2D Dirac materials

**Figure 1:**
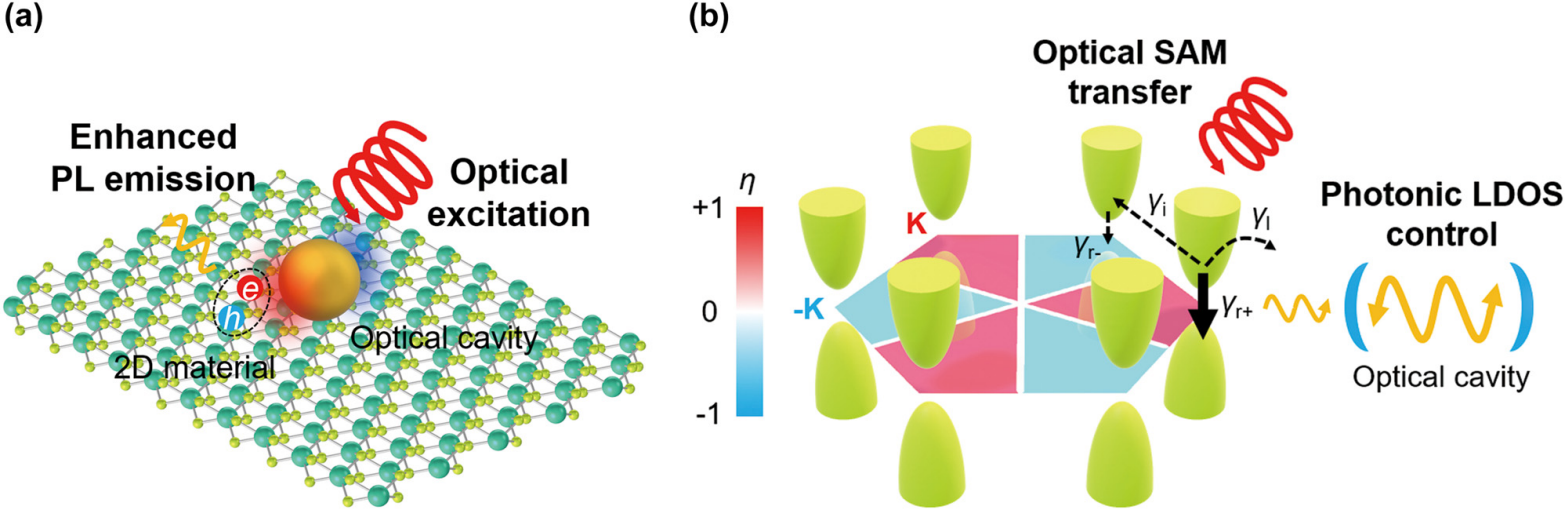
Nanophotonic control of electron behaviors in gapped 2D Dirac materials. (a) Coupling between nanophotonic structures and 2D materials with a honeycomb lattice. (b) Band structures of valleys near *K* and −*K* points (green bowls) in the Brillouin zone (a red and blue colored hexagon) of gapped 2D Dirac materials with the strong SOC. The red and blue colors of the Brillouin zone denote the circular polarization of the emitted photons. If the gapped 2D Dirac materials are coupled to the optical cavity, one of the spontaneous emission rates (*γ*_*r*±_) can be selectively modified by the local photonic density of states (LDOS) control upon the excitation of the pump beam carrying the optical spin angular momentum (SAM). Valley-selective photoluminescence (PL) is determined by the competition of *γ*_*r*±_ and other nonradiative rates (*γ*_*i*_: intervalley scattering rate, *γ*_l_: reservoir leakage rate).

Low-energy properties of quasiparticles are described by fermions obeying the Dirac equation in some materials, such as graphene, transition metal dichalcogenides (TMDs), hexagonal boron nitrides (hBN), Weyl semimetals, and topological insulators [2], [11], [32]. A family of these materials is called Dirac materials. In this article, we focus on light–Dirac matter interaction and its nanophotonic applications. Green bowls in Figure 1 show a schematic drawing for conduction and valence bands near *K* and −*K* points of gapped Dirac materials with a honeycomb lattice. The Dirac materials with the modest (huge) band gap exhibit semiconducting (insulating) properties, while conduction and valence bands with no band gaps forming the Dirac cone result in metallic properties. Note that other effects, e.g., the **L** ⋅ **S** SOC prominent in group-VI TMDs [11], are not included in Figure 1.

Intriguing properties of the 2D Dirac materials appealing to the optics communities are the chiral optical selection rule, i.e., the difference in optical coupling strength according to the handedness of circularly polarized light, and selective excitation of Dirac electrons in a specific extremum of conduction and valence band, namely, valley [2], [11], [32]. A hexagon in Figure 1 corresponds to the Brillouin zone, and its red and blue colors show a degree of circular polarization of photons related to the transition, i.e., absorption and emission, at each valley [12]. Once circularly polarized light carrying spin angular momentum (SAM) excites electrons at a single valley, the excited electrons can be released to the valence bands with photon emission at the rate *γ*_*r*+_ or *γ*_*r*−_ according to SAM of light. In the meantime, some portion of the electrons suffers from the intervalley scattering at the rate *γ*_l_ or scattering to other loss channels at the rate *γ*_l_. When the 2D Dirac materials are coupled to the optical cavity, the photonic local density of states (LDOS) can be modified, resulting in the engineering of the spontaneous emission rate *γ*_*r*±_.

Nanophotonic researchers have paid attention to the chiral optical selection rule of the 2D Dirac materials and the capability of engineering the radiative decay rate *γ*_*r*±_ using the optical cavities. In the following two subsections, we provide a research overview of the coupling of the 2D Dirac materials to the optical cavities.

### 2.1 Control of photoluminescence

**Figure 2:**
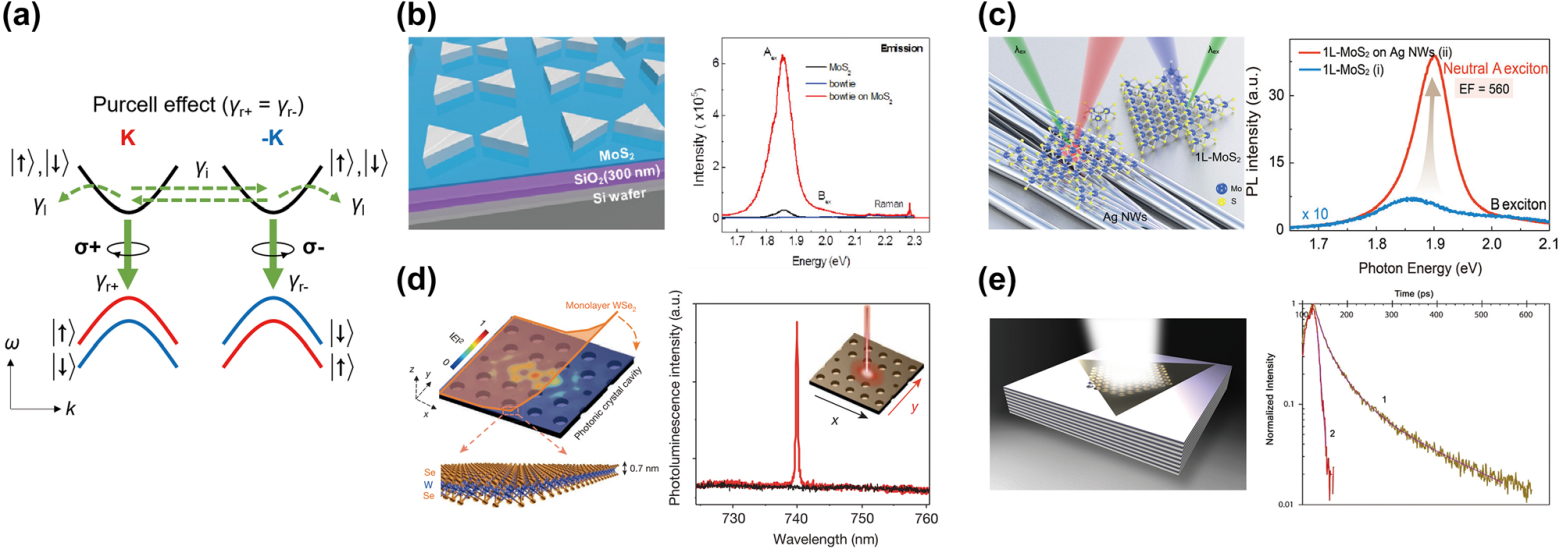
Nanophotonics for the Purcell effect in 2D Dirac materials. (a) Band structure of 2D Dirac materials with strong SOC near *K* and −*K* points. Electron relaxation channels are depicted by green arrows. Emissions via both valleys are enhanced by the Purcell effect. Band diagram is depicted when the optical cavity is linearly polarized resulting in *γ*_*r*+_ = *γ*_*r*−_. (b–d) Nanophotonic structures to enhance PL of 2D TMDs and their enhancement spectra (b: silver bowtie nanoantennas [17], c: silver nanowires [18], and d: the photonic crystal cavity [19]). (e) The hyperbolic metamaterial to enhance PL of monolayer WS_2_ and its exciton lifetime measurement [39]. Reprinted with permission from Ref. [17], copyright 2015 American Chemical Society; Ref. [18], copyright 2018 American Chemical Society; Ref. [19], copyright 2015, Macmillan Publishers Limited; Ref. [39], copyright 2016 American Chemical Society.

Here, we mainly focus on research using group-VI 2D TMDs such as MoS_2_, MoSe_2_, WS_2_, and WSe_2_. They belong to a family of 2D Dirac materials but have strong SOC that distinguishes them from other Dirac materials. Figure 2a illustrates band diagrams near *K* and −*K* points of 2D group-VI TMDs. Because of strong SOC, valence bands split into two according to up and down spins quantized along the out-of-plane direction, while each valley couples to the opposite spin. Excitonic transitions depicted in Figure 2a are called A exciton, exhibiting much higher PL intensity rather than B excitonic transition, which corresponds to the transition between the lower valence band with the opposite spin and the conduction band [13]. Most nanophotonic works have tried to enhance the A excitonic transition using the Purcell effect, the cavity-modification of the spontaneous emission rate.

The Purcell effect has been a foundation of various optical applications, such as lasers [33], single molecule sensing [34], and single photon sources [35] since its discovery in 1946 [36]. As a figure of merit of the optical cavity, the Purcell factor characterizes the performance of the optical cavity to enhance the spontaneous decay rate of quantum emitters. The Purcell factor, defined as $F_{P}=\left(3/4\pi^{2}\right){\left(\lambda /n\right)}^{3}\left(Q/V\right)$ , is an inherent property of a given optical cavity, where *Q* and *V* are the quality factor and the mode volume. *λ*/*n* is the effective emission wavelength, which is normalized by the environment refractive index *n*. The quality factor *Q* describes the temporal confinement of light, i.e., how the cavity resonance persists over time from the initial transient excitation. The mode volume *V* corresponds to the spatial confinement of light, and it describes how much optical energy stored in the cavity is concentrated in the position of the quantum emitter. Using various approaches, the nanophotonics communities have made efforts to achieve large *Q* and small *V*, resulting in a high Purcell factor.

Figure 2b–e are typical examples of the Purcell-enhanced 2D materials. Surface plasmons, a collective oscillation of electrons at the metal/dielectric interface, are one of the ways to get a high Purcell factor because they can confine light in the subwavelength scale, resulting in a small *V*. In Figure 2b, silver bowtie nanoantenna arrays are used to enhance PL of monolayer MoS_2_ [17]. The bowtie nanoantenna structure has sharp tips between two triangular structures, and the lightning rod effect can induce strong local field enhancements at the gap.

A bottom-up approach to constructing plasmonic nanostructures is also possible using plasmonic nanoparticles. Plasmonic nanoparticles are usually synthesized in a solution, and their suspension can be spin-coated onto any substrate or on 2D materials. Figure 2c shows the coupling of 2D TMDs to silver nanowires [18]. Silver nanowires were spin-coated to glass, and monolayer MoS_2_ was wet-transferred on the silver nanowire-coated glass substrate. Silver nanowires have a strong plasmonic response near the A exciton energy of monolayer MoS_2_.

Plasmonic nanostructures can shrink the mode volume *V* in the subwavelength scale, but they suffer from ohmic loss, which draws a limit for the quality factor *Q* (<∼70 for silver and <∼20 for gold) [37]. Dielectric nanostructures can have a larger *Q* factor. A photonic crystal cavity made of dielectric materials can have both large *Q* and small *V*. The photonic crystal cavity in Figure 2d has *Q* = 8000 and 2500 before and after the transfer of monolayer WSe_2_, respectively [19]. Because of its high *Q*, monolayer WSe_2_ coupled to the photonic crystal cavity works as a laser with a low laser threshold.

In addition, hyperbolic metamaterials can enhance the spontaneous emission rate of 2D TMDs [38]. Their optical dispersion has the shape of an open hyperboloid, and emitted photons can couple to the unbounded high-k modes, resulting in the photonic LDOS enhancement. In Figure 2e, a periodic layer of Ag and Al_2_O_3_ thin films form the hyperbolic metamaterials [39]. The hyperbolic metamaterials are perforated by periodic holes, which convert photons at the nonradiative high-k mode into radiation. Monolayer WS_2_ is transferred on the holey hyperbolic metamaterials, and it shows the Purcell factor of ∼20. Ultrafast spectroscopy shows a significant reduction of the lifetime as shown in the right panel of Figure 2e.

### 2.2 Control of valley polarization

**Figure 3:**
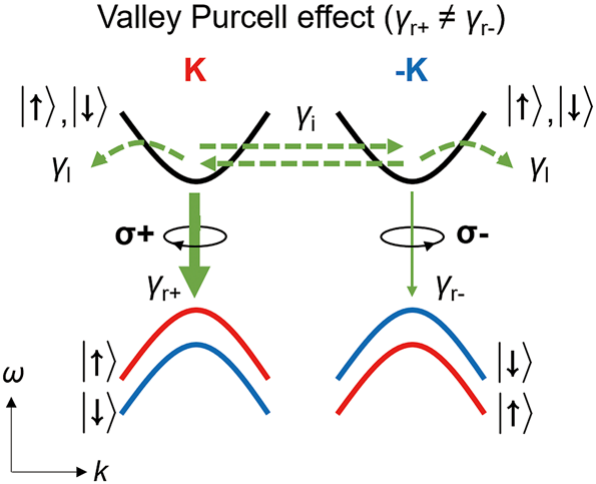
The valley-selective Purcell effect in 2D Dirac materials. Band structure of 2D Dirac materials with strong SOC near *K* and −*K* points. Emission via a single valley can be selectively enhanced by the valley Purcell effect. We introduce the term, the valley Purcell effect, to denote the cavity-driven valley-selective modification of the radiative decay rates *γ*_*r*±_ where *γ*_*r*+_ ≠ *γ*_*r*−_.

Nanophotonic Purcell enhancement for 2D TMDs discussed in Section 2.1 controls excitonic transitions at the band gaps. However, they may not have valley-selectivity (*γ*_*r*+_ ≠ *γ*_*r*−_) because the polarization state of light in the optical cavity was not controlled. Nanophotonic structures can selectively control the spontaneous emission rate in a single valley, i.e., *γ*_*r*+_ ≠ *γ*_*r*−_, as shown in Figure 3. To explain, suppose the optical cavity is fully left-handed circularly polarized. Then, emission with the right-handed circular polarization at the −*K* valley is forbidden, and all the emission occurs only at the *K* valley. In this way, control of valley polarization requires the polarization-selective Purcell enhancement for a single valley.

**Figure 4:**
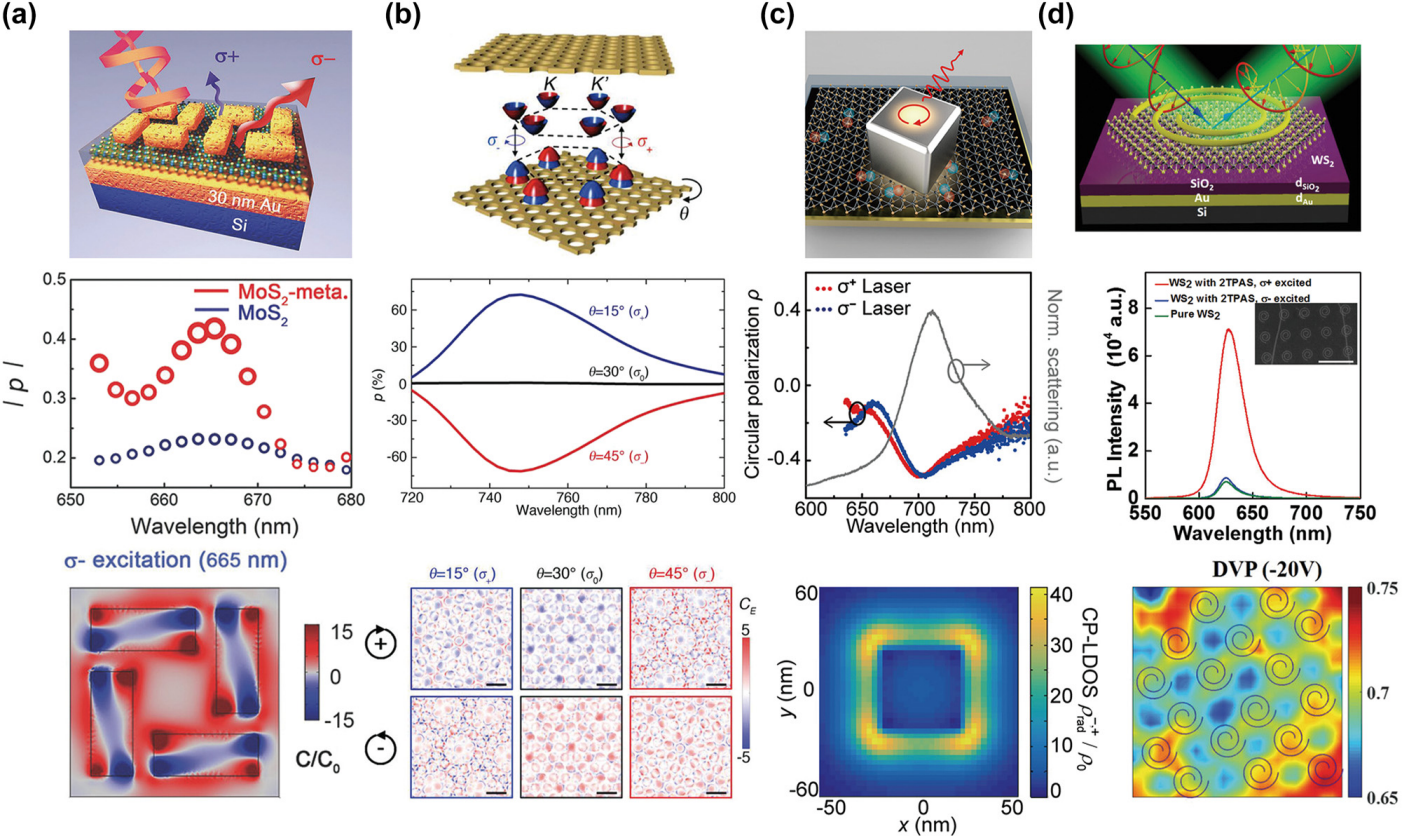
Experimental works on valley-selective PL enhancement. (a–d) Nanophotonic structures to enhance valley-polarized PL of 2D TMDs (top panels), their enhancement spectra (center panels), and chiral near-fields (bottom panels) (a: chiral gold nanorods array [40], b: twisted gold metamaterials [21], c: silver nanocubes gold [41], and d: gold Archimedes spirals [42]). Chirality of near-fields is numerically evaluated by the optical chirality density *C* and the enhanced far-field radiated power in Figure 4a–c, respectively. An accurate way of the chirality evaluation for the near-field is discussed in Sections 3.2 and 3.3. In Figure 4d, the spatial map of the degree of valley polarization (DVP) was measured by dissymmetry of circularly polarized PL emission. Reprinted with permission from Ref. [40], copyright 2018 WILEY-VCH Verlag GmbH & Co. KGaA, Weinheim.; Ref. [21], copyright 2019 WILEY-VCH Verlag GmbH & Co. KGaA; Ref. [41], copyright 2020 American Chemical Society; Ref. [42], copyright 2021 Wiley-VCH GmbH.

A chiral plasmonic nanostructure is a straightforward direction to achieve circularly polarized near-fields. Figure 4a shows an array of chirally arranged gold nanorods [40]. Monolayer MoS_2_ is sandwiched between the gold nanorod array and the gold thin film. The structural chirality of the gold nanorod array provides circularly polarized near-field to monolayer MoS_2_. This work claimed that this chiral near-field enhances the excitonic absorption at a single valley, while it simultaneously quenches that at another valley. For comparison, the degree of circular polarization of bare monolayer MoS_2_ was ±25 %. For monolayer MoS_2_ coupled to the plasmonic nanostructures, the experiments found that the degree of circular polarization in PL is enhanced to 43 % upon the excitation by right-circularly polarized HeNe laser (633 nm), while that is quenched to −23 % [40].

Valley-selective absorption is engineered by plasmonic structures in Figure 4a [40]. On the other hand, the enhancement of the valley-selective emission (*γ*_*r*+_ ≠ *γ*_*r*−_) can be controlled by a chiral nanophotonic environment whose near-fields are circularly polarized [21]. In Figure 4b, monolayer WSe_2_ is sandwiched between two twisted gold nanohole arrays [21]. The coupled system is excited by a linearly polarized laser with center wavelength 532 nm to equally pump the *K* and −*K* valleys. The equal pumping scheme enables the study of the valley-selective modification of the emission rates *γ*_*r*±_. This work found a finite degree of circular polarization in PL of the coupled monolayer WSe2, while the bare monolayer WSe_2_ does not have it upon equal pumping of the two valleys.

A similar approach toward valley-selective emission engineering was tried using plasmonic nanoparticles in Figure 4c [41]. Monolayer MoS_2_ is sandwiched between a silver nanocube and a gold film, forming an atomic thick cavity, namely a nanocube over the mirror (NCOM) structure. Silver nanocubes can be slightly tilted due to their nonperfect shape and rough surfaces. Nanocube tilting results in the broken symmetry of the whole plasmonic structure, lifting the degeneracy in circularly polarized near-fields. The circularly polarized near-fields provide the valley-selective PL emission (*γ*_*r*+_ ≠ *γ*_*r*−_) with a degree of circular polarization up to ∼48 % at room temperature.

Nanophotonically engineered valley-selective emission is also electrically tunable by back-gating. Figure 4d shows gold Archimedes spirals to produce chiral near-fields, supporting the valley-selective PL emission of monolayer WS_2_ [42]. A degree of circular polarization up to 40–50 % was achieved. The plasmon-coupled monolayer WS_2_ device also has a back-gate which dope carriers electrostatically. By the back-gate doping, the degree of circular polarization of plasmon-coupled monolayer WS_2_ is further enhanced to 70 % at room temperature.

### 2.3 Strong coupling of electron and photons

In the previous Sections 2.1 and 2.2, we discuss the nanophotonic control of valley excitons in the weak coupling regime where the Purcell effect modifies the emission rate of a quantum matter. When light–matter interaction becomes significantly large, i.e., the strong coupling regime, photons and electrons in matter form hybrid half-light, half-matter quasiparticles, called polaritons [43].

**Figure 5:**
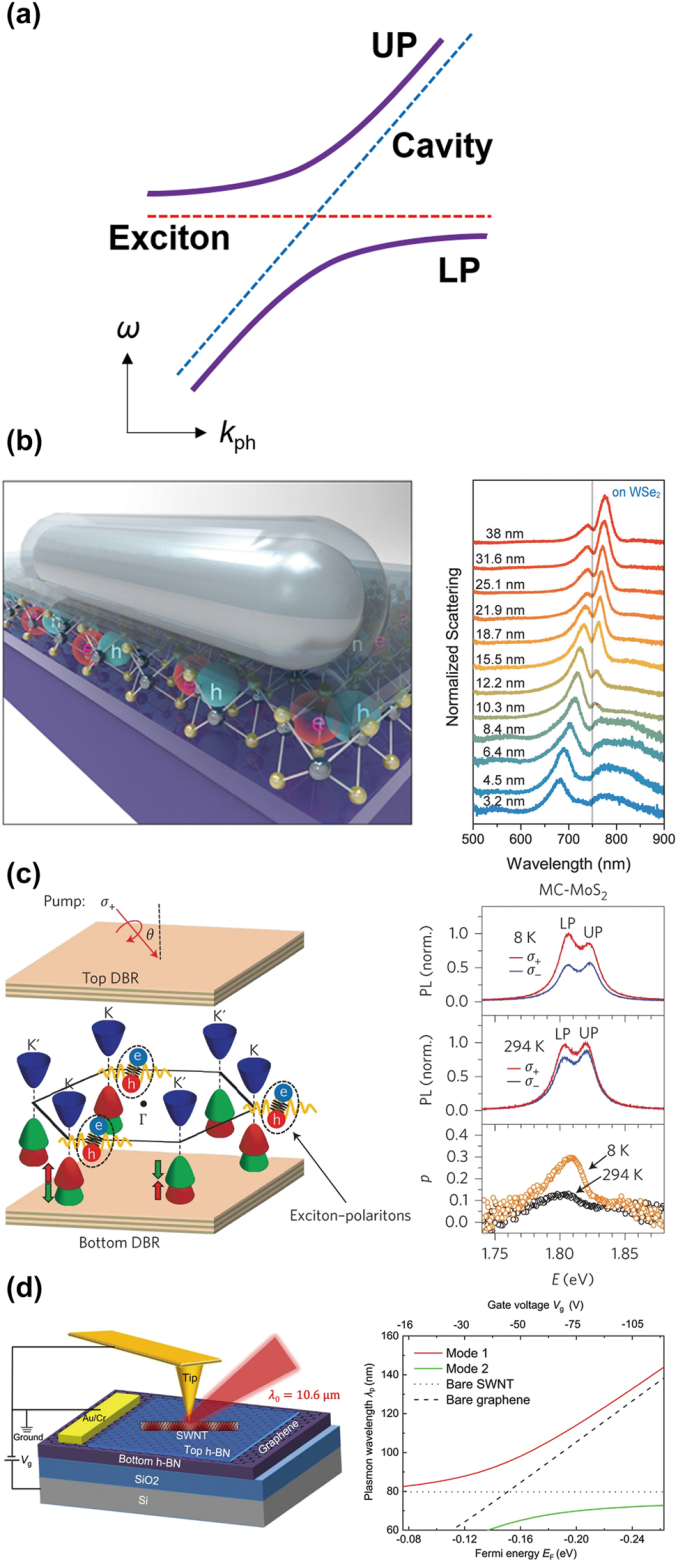
Strong coupling between 2D Dirac materials and nanophotonic structures. (a) Photonic band structure in terms of the photon momentum *k*_ph_ and the energy *ω* (dashed lines: dispersions of a bare cavity and a bare exciton, solid lines: upper and lower polaritons; UP and LP, respectively). (b–d) Nanophotonic structures supporting strong coupling to 2D Dirac materials (left panels) and their strong coupling features (right panels) (b: the silver nanowire Fabry–Perot cavity [23], c: the DBR cavity [25], and d: the metallic single-walled carbon nanotubes [44]). Reprinted with permission from Ref. [23], copyright 2017, American Chemical Society; Ref. [25], copyright 2017, Springer Nature; Ref. [44], copyright 2021, Springer Nature.

A plexciton is a hybrid plasmon–exciton polariton. Silver nanorods of length ∼300 nm in Figure 5b support Fabry–Perot resonance along their rod axis, forming the plexitons [23]. The Fabry–Perot resonance occurs near the exciton energy of monolayer WSe_2_. To tune the Fabry–Perot resonance energy of silver nanorods, a Al_2_O_3_ dielectric coating was successively deposited, while the scattering spectra of nanorods were measured *in situ*. *In situ* scattering measurements confirmed the splitting of a plasmonic peak, a feature of the plexciton formation by the strong coupling.

Valley-contrasting physics can persist even for the 2D Dirac exciton–polaritons in the strong coupling regime. Distributed Bragg reflector (DBR) cavity is one of the best structures to realize the strong coupling because of its huge *Q* factor [24], [25]. In Figure 5c, monolayer MoS_2_ is embedded resonantly by two DBRs. Angle-resolved reflectivity measurements could capture the characteristic dispersion curves of the exciton–polaritons (upper polariton; UP, lower polariton; LP) [25]. To create the circular polarized standing wave inside the DBR cavity, the angle of incidence of the pump beam is carefully chosen. PL emission by the UP and LP also has valley-selectivity with the degrees of circular polarization of ∼20 % and ∼30 % at 8 K, respectively. At room temperature, the valley-selective PL still maintains, i.e., ∼7.5 % and ∼13 % for the UP and LP, respectively. For comparison, bare monolayer MoS_2_ has a degree of circular polarization of ∼40 % and ∼0 % at 8 K and room temperature, respectively.

Strong coupling between two plasmon dispersions from different materials can be possible. Graphene, a 2D semi-metallic Dirac material, hosts plasmons, while single-walled carbon nanotube (SWNT), a 1D metal, also hosts Luttinger liquid plasmons in the infrared frequencies. When the SWNT is coupled to the graphene as shown in the left panel of Figure 5d, two plasmon dispersions form the mixed-dimensional hybrid plasmon dispersion, showing possible evidence of strong coupling in the scanning near-field optical microscopy (SNOM) measurements [44].

## 3 Principles of light–matter interaction in 2D Dirac materials

In Section 2, we have reviewed experimental work for the nanophotonic control of excitonic emission in 2D Dirac materials, especially in 2D group-VI TMDs. What makes 2D Dirac materials distinct from other materials is valley-contrasting physics, including valley-polarized PL. As we have seen in Section 2.2, many experimental works on 2D Dirac materials aimed to control valley-selective excitonic behaviors via the enhancement of the optical chirality density *C*, a measure to characterize the chirality of the complex-valued harmonic electromagnetic field $\left\{\tilde{E},\tilde{B}\right\}$ defined as $C=-\varepsilon_{0}\omega \text{Im}\left({\tilde{E}}^{*}\cdot \tilde{B}\right)$ . These approaches were inspired by recent discoveries that nanophotonics can control chiral light–matter interaction of small chiral molecules with natural optical activity, i.e., circular dichroism enhancement [45] and the cavity modification of circularly polarized emission for small chiral molecules, namely the chiral Purcell effect [22]. Despite the analogy, the physical origins of chiral behaviors in 2D Dirac materials and chiral molecules are different, and thus care should be taken when designing 2D Dirac material–coupled nanophotonic devices. Below, we first discuss the structure of light, i.e., energy/momentum and helicity/spin angular momentum, and then we clarify which component of light interacts with 2D Dirac materials using the gapped Dirac Hamiltonian. Lastly, we discuss common mistakes in designing the nanophotonic structure to control 2D Dirac materials.

### 3.1 Structure of light: spin angular momentum and optical helicity

Light carries momentum and spin angular momentum as physical objects do. Momentum and spin angular momentum transfers lead to changes in energy and helicity, respectively. In this subsection, we introduce mathematical descriptions of the energy-momentum and spin-helicity relations.

The momentum density of light, **g** = **E** × **H**/*c* ^2^, delivers the electromagnetic energy density, $u=\left(E\cdot D+H\cdot B\right)/2$ , by the conservation law, i.e., the Poynting theorem, ∂*u*/∂*t* + ∇ ⋅ **g**/*c* ^2^ = 0 [46]. The momentum density **g** naturally defines the total angular momentum density **j**, the spin angular momentum density **s**, and the orbital angular momentum density ***l*** by the relation, **j** = **r** × **g** = **s** + ***l***. Defining the electric vector potential **E** = −∇ × **C** = −∂**A**/∂*t* in symmetry of the magnetic vector potential **B** = ∇ × **A** = −*μ*_0_∂**C**/∂*t* [47] to follow Berry’s electric–magnetic democracy proposal [48], [49], the spin angular momentum density **s** and the optical helicity density *h* are given by [50], [51], [52]

(1) $$s=\frac{1}{2}\left(\varepsilon_{0}E\times A+\mu_{0}H\times C\right),$$

(2) $$h=\frac{1}{2}\left[\sqrt{\frac{\varepsilon_{0}}{\mu_{0}}}A\cdot \left(\nabla \times A\right)+\sqrt{\frac{\mu_{0}}{\varepsilon_{0}}}C\cdot \left(\nabla \times C\right)\right],$$

in the Coulomb gauge (∇ ⋅ **A** = 0 and ∇ ⋅ **C** = 0), while they follow the conservation law, ∂*h*/∂*t* + *c*∇ ⋅ **s** = 0. For monochromatic fields ( $\tilde{E}=i\omega \tilde{A}$ and $\tilde{H}=i\omega \tilde{C}$ ), the spin angular momentum density and the optical helicity density can be rewritten as

(3) $$s=-\frac{1}{4\omega }\text{Im}\left(\varepsilon_{0}\tilde{E}\times {\tilde{E}}^{*}+\mu_{0}\tilde{H}\times {\tilde{H}}^{*}\right),$$

(4) $$h=\frac{1}{2\omega c}\text{Im}\left(\tilde{E}\cdot {\tilde{H}}^{*}\right),$$

while the conservation law of the spin angular momentum density gives a physical interpretation of the optical helicity, $h=\hat{k}\cdot s$ , i.e., the projection of the spin angular momentum onto the direction of the momentum $\hat{k}$ . In the following Sections 3.2 and 3.3, we discuss how the spin characteristics of light, Eqs. (3) and (4), interact with 2D Dirac materials and chiral molecules, respectively. Note that some works used the optical chirality density *C* rather than the optical helicity density *h*. For monochromatic fields, *h* and *C* differ only by their coefficient, i.e., *h*/*C* = *c*/*ω* ^2^ [52].

### 3.2 Optical response of 2D Dirac materials

Many interesting properties of 2D materials with a honeycomb lattice of the C_3h_ symmetry, e.g., graphene, hBN, and TMD, can be described by the Dirac Hamiltonian derived from a tight-binding model. The gapped Dirac Hamiltonian has the form [2], [11]

(5) $$H=\frac{E_{g}}{2}\sigma_{z}-\hbar \nu_{F}\left(\tau k_{x}\sigma_{x}-k_{y}\sigma_{y}\right),$$

where *E*_*g*_, *v*_*F*_, and *τ* are the bandgap, the Fermi velocity, and the valley index (*τ* = ±1), respectively. *σ*_*x*,*y*,*z*_ are the Pauli matrices, while *k*_*x*,*y*_ are the momentum components. The matrix element of the position operator **r** is given by [2]

(6) $$\left\langle u_{c,k}|r|u_{\nu ,k}\right\rangle =\frac{\left\langle u_{c,k}|\left[H,r\right]|u_{\nu ,k}\right\rangle }{E_{c,k}-E_{\nu ,k}}=R_{k}\left(\tau \hat{x}+i\hat{y}\right),$$

where $\left[H,r\right]=i\hbar \nu_{F}\left(\tau \sigma_{x}\hat{x}-\sigma_{y}\hat{y}\right)$ and *R*_*k*_ is the coefficient determining the oscillator strength. $\left|u_{c,k}\right\rangle$ and $\left|u_{\nu ,k}\right\rangle$ are the eigenvectors of the conduction and the valence bands, respectively. The electric dipole transition matrix element coupled to an arbitrary electric field $\tilde{E}$ is proportional to

(7) $${\left|\left\langle u_{c,k}|r|u_{\nu ,k}\right\rangle \right|}^{2}={\left|R_{k}\right|}^{2}\left\{{\left|{\tilde{E}}_{x}\right|}^{2}+{\left|{\tilde{E}}_{y}\right|}^{2}+2\tau \text{Im}\left({\tilde{E}}_{x}{\tilde{E}}_{y}^{*}\right)\right\}.$$

Interestingly, the last term in Eq. (7) is related to the *z*-component of the electric part of the spin angular momentum density (Eq. (3)), $s_{E,z}=-\left(\varepsilon_{0}/2\omega \right)\text{Im}\left(E_{x}E_{y}^{*}\right)$ as well as the Stokes parameter *S*_3_ = $-2\text{Im}\left(E_{x}E_{y}^{*}\right)$ . Note that the Dirac Hamiltonian is robust and the chiral optical selection rule maintains although some perturbations, such as the **L** ⋅ **S** SOC [2], [11], the Rashba SOC [53], and the magnetic field [54], are added to the gapped Dirac Hamiltonian, Eq. (5).

### 3.3 Analogy and distinction: valley-contrasting physics and natural optical activity

As we have seen in Sections 2.2 and 3.2, 2D Dirac materials obey the chiral optical selection rule, Eq. (7). The chiral optical selection rule of 2D materials is phenomenologically analogous to the natural optical activity of small chiral molecules because both materials selectively respond to circularly polarized light. This analogy inspired early works in the field of nanophotonics to try to control valley polarization in 2D TMDs [21], [41]. In this subsection, we clarify the analogy and distinction between 2D Dirac materials and chiral molecules.

Chiral optical selection rule, Eq. (7), of 2D Dirac materials originate from the C_3h_ rotational symmetry in their orbitals as we discussed in Section 3.2. The corresponding classical picture for 2D Dirac materials is the electric dipole moment **d** = *q* **r** rotating in the *xy*-plane of the 2D material as shown in Eq. (6). This rotating dipole of 2D Dirac materials couples only to the electric field not to the magnetic field because there is no optical magnetism. Circular dichroic (CD) response is also determined by $\text{Im}\left(E_{x}E_{y}^{*}\right)$ .

On the other hand, the natural optical activity of small chiral molecules originates from the interference between the electric dipole (ED) and the magnetic dipole (MD) moments or between ED and the electric quadrupole (EQ) moments [55]. For freely tumbling molecules in a liquid sample, ED–MD interference determines natural optical activity, while the effect of ED–EQ interference cancels out. To describe the optical response of the chiral molecules classically, we can write ED and MD moments in the forms, $\tilde{d}=\tilde{\alpha }\tilde{E}-i\tilde{G}\tilde{B}$ and $\tilde{m}=\tilde{\chi }\tilde{B}+i\tilde{G}\tilde{E}$ , respectively. Electric polarizability, the isotropic electric–magnetic mixed dipole polarizability, and the magnetic susceptibility are given by $\tilde{\alpha }$ , $\tilde{G}$ , and $\tilde{\chi }$ , respectively. Tildes in equations denote complex quantities, e.g., $\tilde{\alpha }=\alpha^{\prime }+i\alpha^{\prime \prime }$ . Then, the absorption rate of molecules is given by [45]

$$A=\frac{\omega }{2}\text{Im}\left(\tilde{d}\cdot {\tilde{E}}^{*}+\tilde{m}\cdot {\tilde{B}}^{*}\right)$$

(8) $$=\frac{\omega }{2}\left(\alpha^{\prime \prime }{\left|\tilde{E}\right|}^{2}+\chi^{\prime \prime }{\left|\tilde{B}\right|}^{2}\right)+G^{\prime \prime }\omega \text{Im}\left({\tilde{E}}^{*}\cdot \tilde{B}\right).$$

The last term in Eq. (8) contains the quantity, $\text{Im}\left({\tilde{E}}^{*}\cdot \tilde{B}\right)$ , that can be related to the optical helicity density, $h=\text{Im}\left(\tilde{E}\cdot {\tilde{H}}^{*}\right)/2\omega c$ (Eq. (4)).

Upon the excitation by the plane wave or the paraxial wave, there is no distinction between CD of valleys and that of natural optical activity. Such waves propagating to the *z*-direction with the circular polarization state satisfy **H**_±_ = ±*i* **E**_±_/*Z*_0_ where the subscript + and – denote right and left circularly polarized light. *Z*_0_ is the impedance of free space. Then, the optical helicity density becomes $h_{\pm }=\text{Im}\left({\tilde{E}}_{\pm }\cdot {\tilde{H}}_{\pm }^{*}\right)/2\omega c=\mp \varepsilon_{0}{\left|{\tilde{E}}_{\pm }\right|}^{2}/2\omega$ , while the spin angular momentum density is written as $s_{\pm }=-\text{Im}\left(\varepsilon_{0}{\tilde{E}}_{\pm }\times {\tilde{E}}_{\pm }^{*}+\mu_{0}{\tilde{H}}_{\pm }\times {\tilde{H}}_{\pm }^{*}\right)/4\omega =-\varepsilon_{0}\text{Im}\left({\tilde{E}}_{\pm }\times {\tilde{E}}_{\pm }^{*}\right)/2\omega =h_{\pm }\hat{z}$ . This also can be seen in the interpretation of the optical helicity, $h=\hat{k}\cdot s=s_{z}$ , derived from the conservation law.

However, for evanescent waves, the equivalence in the spin angular momentum density and the optical helicity density is broken. Therefore, to control the spontaneous emission rate of 2D Dirac materials using the evanescent near fields of the nanophotonic structures, one needs to enhance the out-of-plane component of the spin angular momentum density ( $\propto \text{Im}\left(E_{x}E_{y}^{*}\right)$ ) rather than the optical helicity density ( $\propto \text{Im}\left({\tilde{E}}^{*}\cdot \tilde{B}\right)$ ). On the other hand, chiral molecules respond to the magnetic field **B**, and CD is determined by $\text{Im}\left({\tilde{E}}^{*}\cdot \tilde{B}\right)$ .

Before concluding this section, we emphasize that the orientation factor needs to be included in the calculation of the Purcell enhancement. The semiclassical Purcell theory provides the spontaneous emission rate in the form [56],

(9) $$\gamma =F_{P}\frac{\omega_{0}^{2}}{\omega_{0}^{2}+4Q^{2}{\left(\omega -\omega_{0}\right)}^{2}}\frac{{\left|\tilde{E}\right|}^{2}}{{\left|{\tilde{E}}_{\max}\right|}^{2}}\eta^{2},$$

where ${\left|{\tilde{E}}_{\max}\right|}^{2}$ is the electric field intensity at the maximum point in a given optical cavity. The mode volume *V* in the Purcell factor $F_{P}=\left(3/4\pi^{2}\right){\left(\lambda /n\right)}^{3}\left(Q/V\right)$ can be approximately written as $V=\int \varepsilon {\left|\tilde{E}\right|}^{2}d^{3}r/\varepsilon {\left|{\tilde{E}}_{\max}\right|}^{2}$ . In Eq. (9), the orientation factor of the quantum emitter is defined as $\eta^{2}={\left|\tilde{d}\cdot \tilde{E}\right|}^{2}/\left({\left|\tilde{d}\right|}^{2}{\left|\tilde{E}\right|}^{2}\right)$ . For 2D Dirac materials, the dipole moment is given by $\tilde{d}\propto \tau \hat{x}+i\hat{y}$ . From the expression of the orientation factor, we can draw two important conclusions; (i) the orientation factor naturally shows a valley-selective emission process, i.e.,

(10) $$\eta^{2}=\frac{{\left|\tilde{d}\cdot \tilde{E}\right|}^{2}}{{\left|\tilde{d}\right|}^{2}{\left|\tilde{E}\right|}^{2}}=\frac{{\left|{\tilde{E}}_{x}\right|}^{2}+{\left|{\tilde{E}}_{y}\right|}^{2}+2\tau \text{Im}\left({\tilde{E}}_{x}{\tilde{E}}_{y}^{*}\right)}{2{\left|\tilde{E}\right|}^{2}},$$

For a circularly polarized plane wave, the orientation factor for the 2D Dirac materials, Eq. (10) reduces to $\eta^{2}=\left(1\pm \tau \right)/2$ = 1 or 0. Using Eqs. (9) and (10) for the 2D Dirac materials, we do not need to calculate the chiral Purcell factor written in terms of the optical helicity *h* [22], which enhances the chiral fluorescence caused by the natural optical activity. Instead, one should calculate the out-of-plane component of the spin angular momentum density $s_{E,z}=-\left(\varepsilon_{0}/2\omega \right)\text{Im}\left(E_{x}E_{y}^{*}\right)$ to estimate the valley-selective Purcell effect for 2D Dirac materials. (ii) Without taking the orientation factor, Eq. (10), into account, one may overestimate the Purcell factor for the 2D material coupling.

## 4 Perspectives

Valley-contrasting physics is appealing to nanophotonics and optoelectronic applications as we have seen in Section 2. It has been known that valley-contrasting physics in monolayer Dirac materials persist when the external perturbations, e.g., the optical field, the magnetic field, the electrostatic gating, the spin injection, and the magnetic proximity effect. Also, the interlayer excitons in 2D heterostructures inherit valley-contrasting properties in a single monolayer. This robustness of valley-contrasting physics in 2D Dirac materials and their heterostructures implies that there is room for nanophotonic coupling. Here, we list typical examples of external perturbations and novel excitons exhibiting valley-contrasting physics and the chiral optical selection rule. It is noteworthy that valley-contrasting physics in 2D Dirac materials is closely related to symmetry breaking. One can find a comprehensive review of the symmetry-breaking engineering in 2D materials in Ref [57].

### 4.1 Magnetic field: the valley Zeeman effect and the spin mixing effect

**Figure 6:**
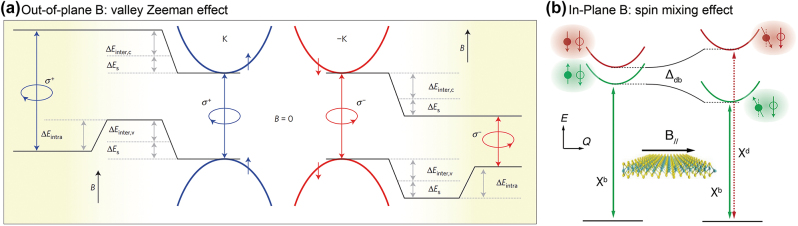
Effect of the magnetic field on 2D Dirac materials. (a) The valley Zeeman effect for the out-of-plane magnetic field [58]. (b) The spin mixing effect for the in-plane magnetic field [60]. Reprinted with permission from Ref. [58], copyright 2015, Macmillan Publishers Limited.; Ref. [60], copyright 2019, IOP Publishing Ltd.

*K* and −*K* valleys of gapped 2D Dirac materials are degenerate in energy (e.g., Figures 1a, 2a, and 3), while they possess opposite spins governed by time-reversal symmetry. External magnetic fields (B) can break time-reversal symmetry, lifting the degeneracy of the two valleys, i.e., the valley-Zeeman effect. The out-of-plane B field can change energies of conduction and valence bands of the two valleys differently as shown in Figure 6a [58]. In the valley Zeeman effect, the spin states of each band are preserved, and the chiral optical selection rules are maintained, while two opposite circularly polarized PL emission from *K* and −*K* valleys have different energies. On the other hand, the in-plane B field can brighten the dark excitons, which have a larger *g* factor compared to the bright excitons [59]. The bright and dark excitons correspond to the spin-preserving radiative transition and the spin-forbidden transition, respectively. The in-plane B field can mix spins in conduction band electrons, redistributing some oscillator strength of bright excitons to dark excitons (Figure 6b) [60]. Then, the dark excitonic transition can emit PL.

### 4.2 Optical field: the optical Stark effect, the Bloch–Siegert effect, and the Floquet engineering

**Figure 7:**
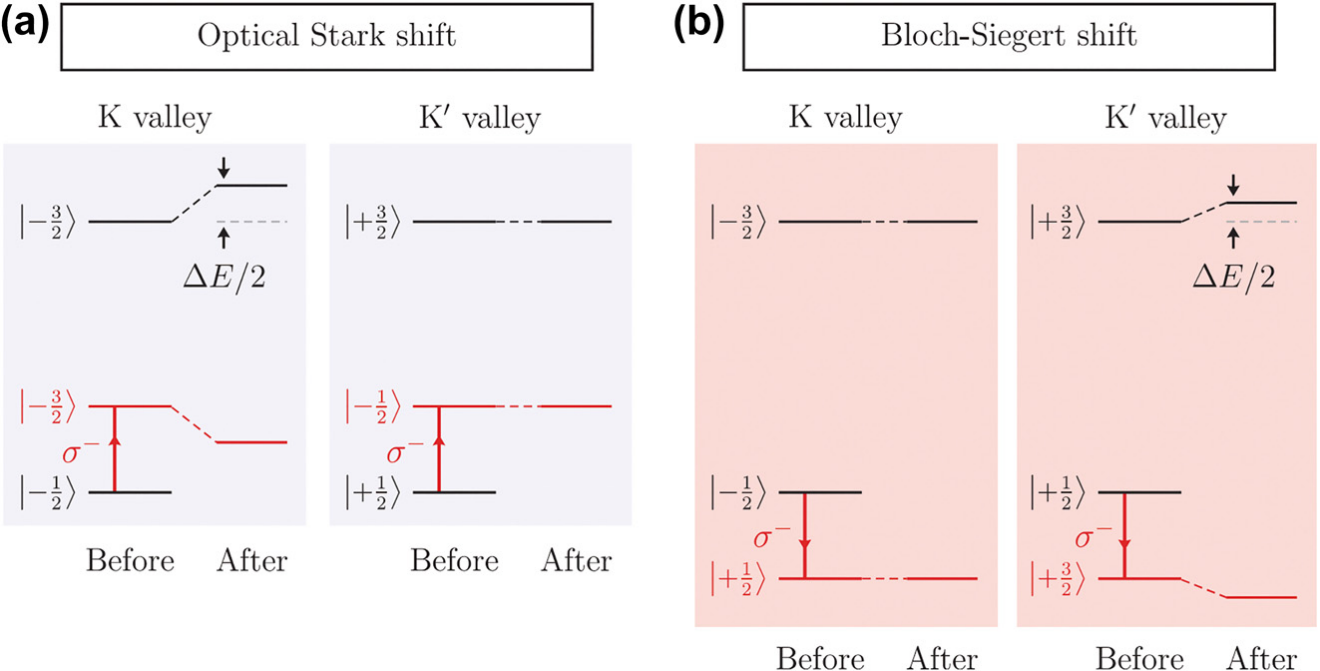
Effect of the intense optical field on 2D Dirac materials. Band diagrams and possible radiative transitions (red arrows) for (a) the optical Stark effect and (b) the Block–Siegert effect [62]. Reprinted with permission from Ref. [62], copyright 2017 American Association for the Advancement of Science.

Quantum interaction between photons and a given electronic system can induce a series of photon–electron hybrid states, called the Floquet states [61], [62]. In the leading order, a pair of the original two-level electronic system with the energy *E*_0_ split into two Floquet pairs: one with the energy smaller than the original pair and another with that larger than the original pair. State repulsion between the former (latter) Floquet state and the original state is called the optical Stark effect (the Bloch–Siegert effect) with the energy shift $\Delta E_{OS}\propto I/\left(E_{0}-\hbar \omega \right)$ ( $\Delta E_{BS}\propto I/\left(E_{0}+\hbar \omega \right)$ ). *I* and *ℏω* are the intensity of light and the photon energy, respectively. The optical Stark effect and the Bloch–Siegert effect in the 2D Dirac materials also inherit the valley-selectivity of the original material. Figure 7 shows energy diagrams and possible radiative transitions for the two effects [62]. For the pump with the helicity *σ* ^−^ (*σ* ^+^), the optical Stark shift and the Bloch–Siegert shift occur only at the *K* and *K*′ valleys (the *K*′ and *K* valley), respectively. Strong interaction between the electronic states and the Floquet states also suggests light-driven engineering of the electronic structure, called the Floquet engineering [61], [63], [64]. Recently, the experimental realization of a mid-infrared pump/visible probe of Floquet-engineered excitons in monolayer WS_2_ has been reported [64].

### 4.3 Interlayer excitons and Moire superlattice

**Figure 8:**
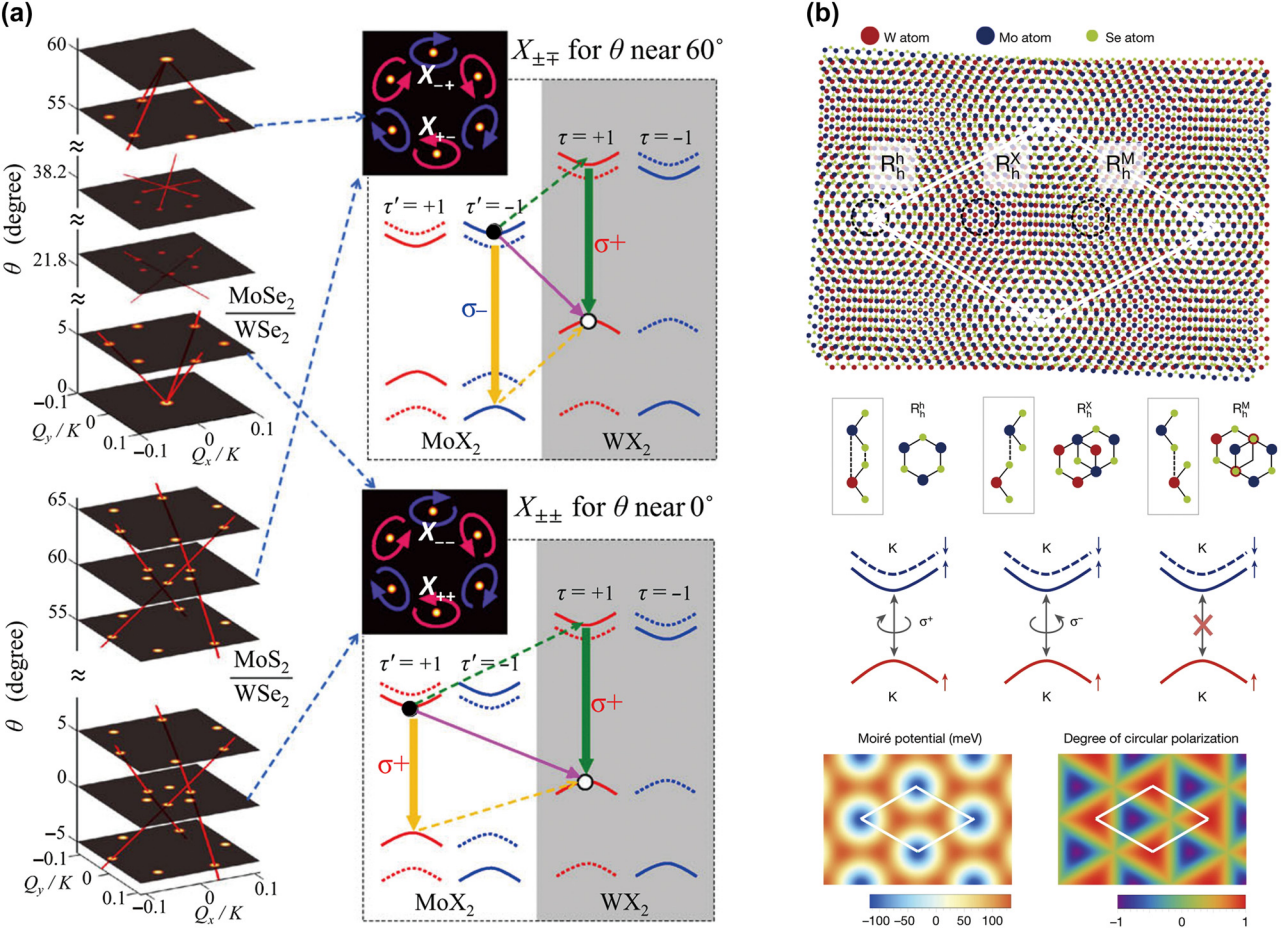
Interlayer excitons and Moire phenomena in 2D heterostructures. (a) Light cones of MoX_2_/WX_2_ heterobilayers (X: Se or S) with different twist angles *θ* [65]. Interlayer excitonic transitions and band diagrams for *θ* ∼ 60°. (b) Moire superlattice of a MoSe_2_/WSe_2_ heterostructure with a small twist angle [68]. Possible transitions at three atomic sites are depicted. Moire potential of the interlayer exciton transition and the optical selection rule for the *K* valley excitons are plotted. Reprinted with permission from Ref. [65], copyright 2015, American Physical Society; Ref. [68], copyright 2019, Springer Nature.

A 2D heterostructure, a vertical stack of different 2D materials, hosts interlayer excitons between neighboring monolayers. The interlayer exciton also has the valley-contrasting physics as the constituent monolayers do. For example, Figure 8a shows a theoretical prediction for light cones of MoX_2_/WX_2_ heterobilayers (X: Se or S) with different twist angles *θ*, suggesting the valley-selective transitions with the elliptical PL polarizations at the main light cones [65]. It also has been known that the spatially spread interlayer excitons have significantly weak oscillator strength compared to the intralayer exciton [66], [67]. Therefore, we expect that nanophotonic Purcell enhancement of the interlayer excitonic dipole is a promising way to excite and detect the interlayer exciton.

Also, a quasiperiodic atomic pattern by lattice mismatch and/or twisting between monolayers forms the so-called Moire interlayer excitons. The optical selection rule for the circular polarizations differs according to the sites of the interlayer atomic registry (Figure 8b [68]). The Moire interlayer exciton can support many quantum phenomena and rich physics, while the optical, electrical, and magnetic properties of the Moire interlayer excitons can be engineered by constitutions, twist angle, temperature, and external perturbations (e.g., electric and magnetic field).

## 5 Conclusions

Before concluding, it is worth mentioning that the gapless Dirac materials, e.g., 2D graphene and 1D metallic single-walled carbon nanotubes (SWNT), host plasmons with huge Q factors in infrared frequencies [69], [70], [71], [72]. 2D plasmons in graphene have been of huge interest in the past decade, and many works have opened a field of “graphene plasmonics” [73], [74], [75], [76]. Recently, the Fizeau drag in 2D plasmons of biased graphene, changes in the plasmon wavelength according to the driving current direction, has been experimentally demonstrated by infrared real-space imaging [77], [78]. The Fizeau drag effect can introduce nonreciprocity in 2D plasmons, and this novel concept can provide new possibilities in graphene plasmonics [79], [80]. Despite huge successes in graphene plasmonics, 1D plasmons in metallic SWNT have not brought enough attention from nanophotonics. Electronic behaviors in 1D metals are completely different from those in higher dimensional metals. Strong electron–electron interaction in 1D requires electrons to be a strongly correlated quantum matter, the so-called Luttinger liquid. 1D Luttinger liquid possesses exotic properties such as the spin-charge separation [81], the forbidden back-scattering [82], and the power-law scaling in the tunneling conductance [83], [84]. Especially, 1D Luttinger liquid plasmons have the highest Q factor among the reported plasmons in various metals because the quasiparticle excitation including thermally excited phonons is forbidden in the Luttinger liquid [69], [72]. The nonlinear Luttinger liquid has also been experimentally demonstrated in semiconducting carbon nanotubes at the infrared frequency, and their plasmonic properties, i.e., the plasmon wavelength and the Q factor, can be tuned by electrostatic gating [85]. Also, it has recently been suggested that electrically driven photon emission may be possible in metallic SWNTs by the electron injection to the SWNTs and the subsequent plasmon-to-photon conversion by the nanoantenna in their vicinity [86], [87]. Mixed dimensional heterostructures, e.g., 2D graphene/1D carbon nanotube, also offer nanophotonic tunablity and hybrid properties [44]. Exotic quantum and optical properties of 1D Luttinger liquid promise a huge possibility in nanophotonic applications.

In this article, we reviewed the recent works on nanophotonics-controlled 2D materials. Nanophotonic structures can enhance the overall intensity of PL (Section 2.1) and/or the circularly polarized PL emitted by a single valley of the 2D materials (Section 2.2). To offer a design guide for nanoresonators for the 2D Dirac materials and correct misconceptions on the chiral light–matter interaction, we discussed a principle of chiral optical selection rule in 2D Dirac materials and compared it to circular dichroism of chiral molecules with natural optical activity (Section 3). We provided perspective on the externally perturbed 2D material properties that can be enhanced by the resonant nanophotonic structures (Section 4). We believe our review can promote and guide future efforts on the nanophotonic control of electron behaviors in 2D Dirac materials.
